# Supplementary material for: Comparative Mortality and Adaptation of a Smurf Assay in Two Species of Tenebrionid Beetles Exposed to Bacillus thuringiensis
Source: Insects. 2020 Apr 24;11(4):261. doi: 10.3390/insects11040261 (PMC7240575; doi:10.3390/insects11040261)
Supplement: Supplementary file 1 [file insects-11-00261-s001.zip › Zanchi et al. Supplement Figures S1 and S2.pdf]

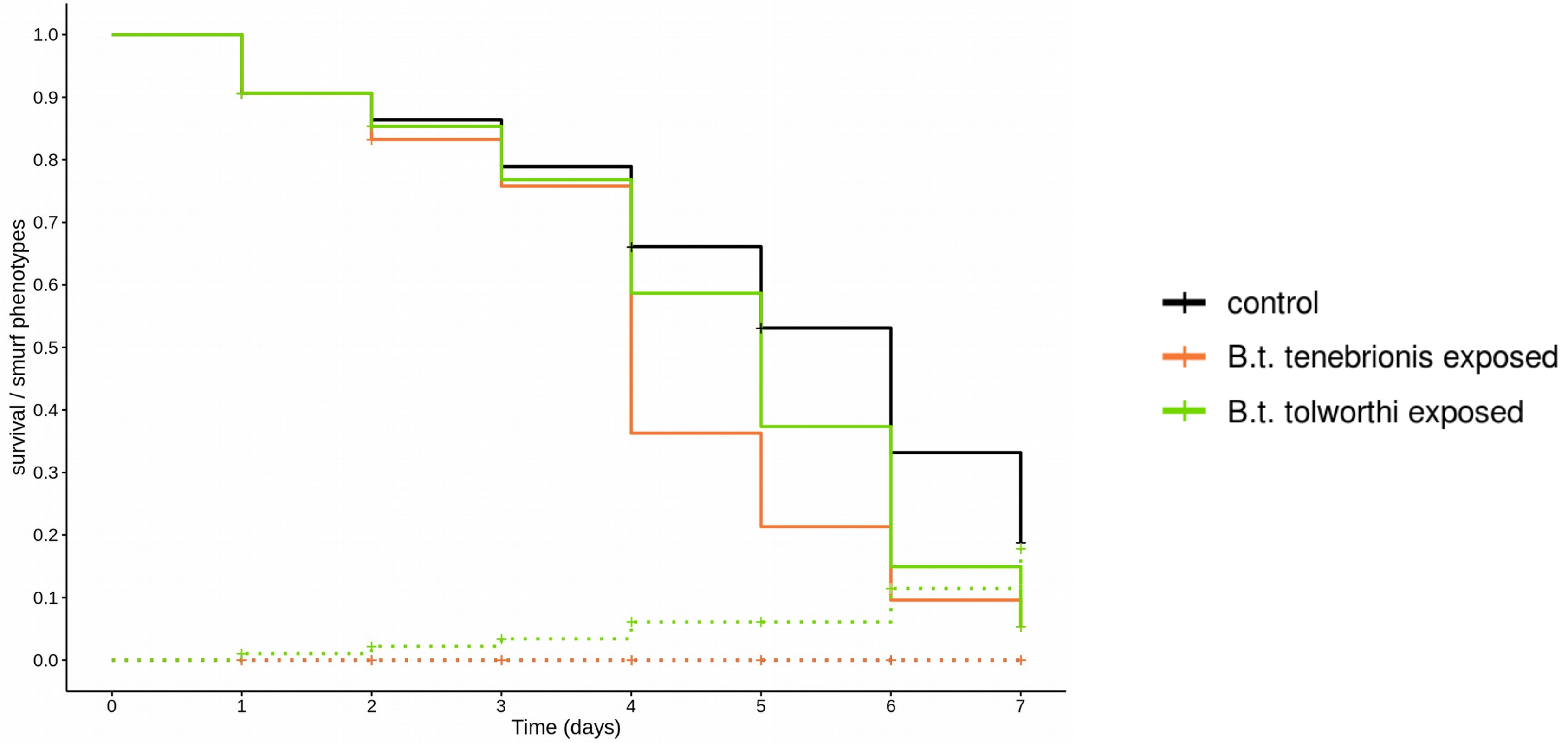

Figure S1: Survival of 13-days old *T. castaneum* larvae during exposure to *B. thuringiensis* var. *tenebrionis* and var. *tolworthi*. Kaplan-Meier curve representing the survival (plain descending lines) and the incidence of Smurf phenotypes (dashed ascending lines) over 7 days in larvae of *Tribolium castaneum* which were 13 days old after egg lay at the beginning of the experiment, exposed to a control diet (in **black**), 2.5 x 10^9 CFU/mL of *B. thuringiensis* var. *tenebrionis* (in **orange**) and 2.5 x 10^9 CFU/mL of *B. thuringiensis* var. *tolworthi* (in **green**) spores and crystals mix. The exposure treatment has a significant effect on both survival ($X^2_{2,247} = 20.1$; $p < 0.001$) and the occurrence of smurf phenotype ($X^2_{2,247} = 19.24$; $p < 0.001$). However, the control treatment shows a high mortality, indicating that 13days old larvae die of handling.

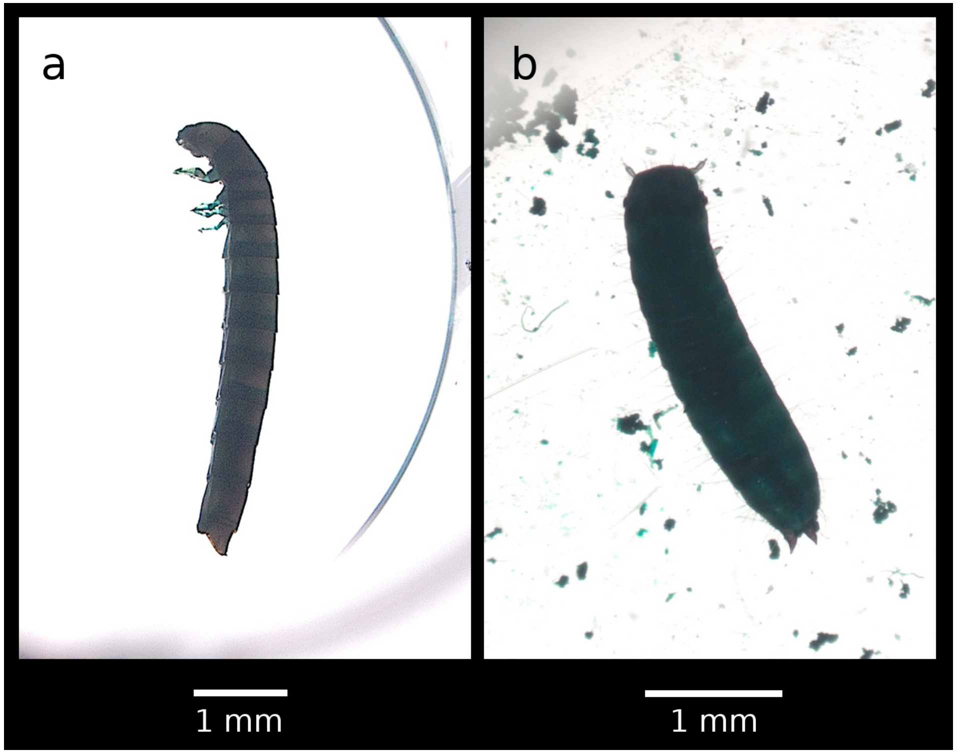

Figure S2: Appearance of the Smurf phenotype 48 hours after death. Larvae of **a)** *Tenebrio molitor* and **b)** *Tribolium castaneum* 48 hours after having shown the Smurf phenotype upon ingestion of $2.5.10^9$ CFU/mL of *Bacillus thuringiensis* spores and crystals mix. The melanization of the larvae makes it hard to distinguish the characteristic blue hue in the hemolymph of dead larvae. We therefore chose to record the occurrence of Smurf phenotypes only in live of newly dead larvae (dead since 24 hours or less, according to our sampling procedure, see Materials & Methods).
